# SARS-CoV-2 Seroprevalence and Profiles Among Convalescents in Sichuan Province, China

**DOI:** 10.3389/fpubh.2021.716483

**Published:** 2021-10-26

**Authors:** Lijun Zhou, Cheng Li, Huiping Yang, Heng Yuan, Ming Pan, Xiuwei Cheng, Chongkun Xiao, Xiaoyan Su, Yuanfang Zhu, Jianan Xu, Jianxiang Tang, Xunbo Du, Huanwen Peng, Xiao Chen, Tao Huang, Hongxiu Liao, Deqiang Xian, HaoZhou Wang, Wenwu Liu, Ping Zhou, Zhengdong Zhang, Juan Liu, Xianping Wu, Tao Zhang

**Affiliations:** ^1^Sichuan Center for Disease Control and Prevention, Chengdu, China; ^2^Department of Epidemiology and Health Statistics, West China School of Public Health and West China Fourth Hospital, Sichuan University, Sichuan, China; ^3^Lu County Center for Disease Control and Prevention, Luzhou, China; ^4^Gulin County Center for Disease Control and Prevention, Luzhou, China; ^5^Chengdu Center for Disease Control and Prevention, Chengdu, China; ^6^Dazhou Center for Disease Control and Prevention, Dazhou, China; ^7^Nanchong Center for Disease Control and Prevention, Nanchong, China; ^8^Guangan Center for Disease Control and Prevention, Guangan, China; ^9^Panzhihua Center for Disease Control and Prevention, Panzhihua, China; ^10^Luzhou Center for Disease Control and Prevention, Luzhou, China; ^11^Mianyang Center for Disease Control and Prevention, Mianyang, China; ^12^Suining Center for Disease Control and Prevention, Suining, China; ^13^Yibin Center for Disease Control and Prevention, Yibin, China; ^14^Zigong Center for Disease Control and Prevention, Zigong, China; ^15^Neijiang Center for Disease Control and Prevention, Neijiang, China

**Keywords:** seroprevalence, SARS-CoV-2, associated factors, convalescents, duration

## Abstract

**Objectives:** To explore and understand the SARS-CoV-2 seroprevalence of convalescents, the association between antibody levels and demographic factors, and the seroepidemiology of convalescents of COVID-19 till March 2021.

**Methods:** We recruited 517 voluntary COVID-19 convalescents in Sichuan Province and collected 1,707 serum samples till March 2021. Then we reported the seroprevalence and analyzed the associated factors.

**Results:** Recent travel history was associated with IgM levels. Convalescents who had recent travel history were less likely to be IgM antibody negative [OR = 0.232, 95% CI: (0.128, 0.420)]. Asymptomatic cases had, approximately, twice the odds of being IgM antibody negative compared with symptomatic cases [OR = 2.583, 95% CI: (1.554, 4.293)]. Participants without symptoms were less likely to be IgG seronegative than those with symptoms [OR = 0.511, 95% CI: (0.293, 0.891)]. Convalescents aged 40–59 were less likely to be IgG seronegative than those aged below 20 [OR = 0.364, 95% CI: (0.138, 0.959)]. The duration of positive IgM antibodies persisted 365 days while the IgG persisted more than 399 days.

**Conclusions:** Our findings suggested that recent travel history might be associated with the antibody levels of IgM, while age could be associated with the antibody levels of IgG. Infection type could be associated with both antibody levels of IgM and IgG that declined quicker in asymptomatic cases.

## Introduction

The global pandemic of coronavirus disease 2019 (COVID-19), an emerging infectious disease seeding from severe acute respiratory syndrome coronavirus 2 (SARS-CoV-2), has posed a serious threat to public health (1). The epidemiological and serological characteristics of patients with COVID-19 have been reported explicitly (2), while few have paid attention to convalescents.

Antibody response is crucial in eliminating viral infection (3), and the seroprevalence of specific serum antibodies including immunoglobulin M (IgM) and immunoglobulin G (IgG) against SARS-CoV-2 can provide immune protection. Understanding the seroprevalence dynamic of SARS-CoV-2 assists in assessing the immunologic levels of convalescents and predicting the potential immune protection (4). In the case of SARS-CoV-2, IgM responses firstly against viral infection, while IgG production lags behind IgM but produces a more durable immunity (5), similar to the response process in coronavirus infections such as severe acute respiratory syndrome coronavirus (SARS) and middle east respiratory syndrome coronavirus (MERS) (6). Previous studies have reported that specific IgM antibodies last only for 13 weeks in the body (7), while IgG antibodies are more longstanding with an average of 2 years (8). The decline of IgM, as an indicator of virus clearance, suggests convalescents' robust immunity against re-infection with positive antibody, while the reduction of IgG prompts serious concerns on the robustness and persistence of immunity after recovery (9). Studying the seroprevalence of these antibodies is primary for developing vaccine and immunity strategies. Previous studies (10–13) have explored the seroprevalence of these antibodies from diversified perspectives, such as the accuracy of serological tests, immunological memory, and molecular findings. However, associated factors and the duration of positive antibodies still require to be updated.

As the number of patients recovering from the SARS-CoV-2 continues to rise, the duration of individual serological responses has attracted public attention (14). Most of the previous researches mainly focused on the acute response within several weeks after clinical onset in SARS-CoV-2. As the number of convalescents re-infected with SARS-CoV-2 started to escalate, testified by those initial symptomatic cases re-infected with SARs-CoV-2 reported in several countries (15), clarifying the antibody response duration to the virus after infection is of paramount significance. Additionally, understanding whether the demographic factors (such as age, gender, recent travel history, and infection type) were associated with serological responses during SARS-CoV-2 infection is also vital. Studying the associated factors contributes to our understanding of the body's response to SARS-CoV-2 at different stages. However, as most studies focused on molecular and cellular reports (16, 17), researches from the perspective of public health were few, such as profiles, associated factors and so on.

Therefore, we focused on the dynamics of seroprevalence to special antibodies and the factors associated with antibody results. By collecting and analyzing the serological level information on 517 convalescents of SARS-CoV-2 in Sichuan Province, this study assessed whether seroprevalence was associated with demographic factors, such as gender, age, infection type (symptomatic cases or asymptomatic cases), and recent travel history (cases with or without recent travel history). Meanwhile, we described the dynamic serum changes and durability of convalescents of SARS-CoV-2. Our study aimed to profile the demographic features of convalescents and explore the relationship between characteristics and humoral responses, which could give insights into the humoral immune responses among the convalescents. Additionally, in this study, we observed the difference between symptomatic cases and asymptomatic cases, which could offer some clues for the prevention and therapy for COVID-19 patients.

## Methods

### Study Design

This study is a retrospective cohort study including 517 convalescents in Sichuan Province as of March 12, 2021. All subjects voluntarily joined the serological research and gave their informed consents. We collected 1,707 serum samples and demographic characteristics of 517 convalescents. The variables introduced were gender, age groups (<20 years old, 20–39 years old, 40–59 years old, and ≥60 years old), recent travel history (cases with or without recent travel history), infection type (symptomatic or asymptomatic), and antibody results (positive or negative). The antibody results were outcomes of interest.

### Data and Specimen Collection

Data were collected by the Sichuan Center for Disease Control and Prevention, consisting of demographic characteristics of the 517 convalescents and their longitudinal antibody results (1,707 serum samples). Specimens were collected from June 23, 2020, to March 12, 2021, based on voluntary informed consent of COVID-19 convalescents. Participants all met the criteria according to the *Diagnosis and Treatment Protocol for Novel Coronavirus Pneumoni* (Trial Version 8 and subsequent versions) released by the National Health Commission & State Administration of Traditional Chinese Medicine. Consenting individuals who were diagnosed with COVID-19 and not vaccinated were asked to do serology testing. We excluded individuals who were unable to go to designated locations for the blood draw and those who had severe complications and those on immunity inhibitors. Written informed consent was provided by all study participants or their parents, and parental permission was obtained before collecting serum samples. The interval between two serum collections was not less than 30 days, and the same batch of serum samples was detected simultaneously and operated by the same laboratorians. All 1,707 serum samples were detected by the Institute of Microbiology and Analysis. The SARS-CoV-2 IgM and IgG antibodies were detected using a 2019-nCoV IgG/IgM detection kit (Maccura Biotechnology Co., Ltd, Sichuan, China). IgM and IgG were observed to have antibody responses against RBD proteins, which could neutralize the virus.

### Detection of IgG and IgM

Non-anticoagulant specimens (intravenous blood collection) were collected for all subjects, 3 mL for children (aged below 5 years), and 5 mL for others. Serum samples were collected, loaded into sealed bags following Class A transport packaging, refrigerated, and transported to the local CDC laboratory for serum separation. The isolated serum was stored in a 1.5 mL frozen deposit tube at −20 degrees C. The Maccura 1,000 fully automated luminescent immunoanalystator (base fluid lot number: 0520153; reagent lot number: 0520031,0520032; reaction cup lot number: 0720582) was utilized to test serum by the principle of direct chemical luminescence immune analysis.

### Ethical Approval

All participants assented to informed consent before participation, and this study was conducted under Good Clinical Practice (GCP). This study was performed in compliance with all relevant ethical regulations. The protocol for human subject studies was approved by the Sichuan Center for Disease Control and Prevention (SCCDCIRB-2020-007).

### Statistical Analysis

Descriptive statistics were utilized to summarize the demographic characteristics of the cohort and significant study outcome variables. Median and Inter-Quartile Range (IQR) were used to describe age. Then frequency and composition ratio were used for categorical variables. Furthermore, the Chi-squared test or Fisher's exact test was applied for comparing categorical variables. Finally, multivariable logistic regression was adopted to calculate odds ratios and 95% confidence interval. The Kaplan-Meier method was applied for the seroprevalence changes, and the log-rank test was used to calculate the difference for positive rates of specific antibodies IgM and IgG over time. All analyses were performed by Stata 16.0 software, and the *p*-value <0.05 in this paper was considered statistically significant.

## Results

### Demographic and Clinical Characteristics

By March 12, 2021, a total of 517 participants (363 males; 154 females) were recruited. A descriptive analysis of the 517 convalescents with SARS-CoV-2 infections was detailed in Tables 1, 2. The median age of participants was 47 (IQR = 33–57) years. For serology results, 125 cases were IgM positive and 392 cases were IgM negative, while 417 cases were IgG positive and 100 cases were IgG negative. For recent travel history, those who had recent travel history occupied the majority of positive antibodies cases (IgM: 102 cases; IgG: 266 cases). Asymptomatic cases occupied 31.33% (162 cases), while symptomatic cases accounted for 68.67% (355 cases). The majority of the COVID-19 cases with positive IgG antibodies ranged from 20 years to 39 years old (210 cases), similar to those with positive IgM antibodies (59 cases).

**Table 1:** Demographic and clinical characteristics of IgM.

	**Total** **(No.)**	**IgM No. (%)**					
		**Negative**	**Positive**	**χ^**2**^**	**uOR**	**95%CI**	***P***
	517	392 (75.82)	125 (24.18)				
Gender				13.32	0.393	[0.235, 0.657]	<0.01
Female	154	133 (86.36)	21 (13.64)				
Male	363	259 (71.35)	104 (28.65)				
Recent travel history				24.34	0.301	[0.183, 0.493]	<0.01
With	326	224 (68.71)	102 (31.29)				
Without	191	168 (87.96)	23 (12.04)				
Infection type				6.12	1.799	[1.125, 2.877]	0.014
Asymptomatic	162	134 (82.72)	28 (17.28)				
Symptomatic	355	258 (72.68)	97 (27.32)				
Age (years)				5.19	0.902	[0.689, 1.181]	0.452
<20	24	21 (87.50)	3 (12.50)				
20–39	256	197 (76.95)	59 (23.05)				
40–59	183	130 (71.04)	53 (28.96)				
≥60	54	44 (81.48)	10 (18.52)				

*Values are n (%). Groups were compared using the Chi-squared test*.

*IgG, immunoglobulin G; IgM, immunoglobulin M; uOR, unadjusted odds ratio; CI, confidence interval*.

**Table 2:** Demographic and clinical characteristics of IgG.

	**Total** **(No.)**	**IgG No. (%)**					
		**Negative**	**Positive**	*****χ^2^*****	**uOR**	**95% CI**	***P***
	517	100 (19.34)	417 (80.66)				
Gender				0.46	1.184	[0.727, 1.929]	0.498
Female	154	27 (17.53)	127 (82.47)				
Male	363	73 (20.11)	290 (79.89)				
Recent travel history				0.50	0.852	[0.544, 1.332]	0.481
With	326	60 (18.40)	266 (81.60)				
Without	191	40 (20.94)	151 (79.06)				
Infection type				5.02	0.558	[0.333, 0.934]	0.026
Asymptomatic	162	22 (13.58)	140 (86.42)				
Symptomatic	355	78 (21.97)	277 (78.03)				
Age (years)				3.36	0.942	[0.701, 1.266]	0.692
<20	24	8 (33.33)	16 (66.67)				
20–39	256	46 (17.97)	210 (82.03)				
40–59	183	35 (19.13)	148 (80.87)				
≥60	54	11 (20.37)	43 (79.63)				

*Values are n (%). Groups were compared using the Chi-squared test*.

*IgG, immunoglobulin G; IgM, immunoglobulin M; uOR, unadjusted odds ratio; CI, confidence interval*.

### Specific Antibodies IgM and IgG Levels

The levels of different antibodies in 517 patients infected with SARS-CoV-2 were further described in Tables 1, 2. IgM and IgG seroprevalences were diverse among different convalescents. The majority of patients tended to be IgG positive (417 cases) and IgM negative (392 cases). And the proportion of IgM positive in infected females (13.64%) was significantly lower than in infected males (28.65%), but the proportion of IgG positive was similar between both genders. The proportion of cases with recent travel history was 31.29% for positive-IgM and 81.60% for positive-IgG. Further, a discrepancy in seroprevalence was observed among age subgroups (Table 2), with the peak in the subset of people aged 40–59 (IgM, 28.96%) and 20–39 years (IgG, 82.03%). The lowest positivity rates (IgM, 12.50%; IgG, 66.67%) were observed in groups aged below 20 years. The antibody levels of IgM showed significant differences in different genders and travel histories (*P* < 0.01). We also observed a statistically significant difference in the antibody levels of IgG between different infection types (*P* = 0.026).

### Multivariable Logistic Regression Analysis for IgM

In Table 3, the antibody level of IgM antibody (positive or negative) was taken as the dependent variable, while gender and infection type as the independent variables. Female, cases without recent travel history, and symptomatic cases were regarded as the reference, respectively. Different age groups were computed as dummy variables, while the age group younger than 20 years was the reference group. Previous studies had found that gender and age are related to the outcome levels of antibodies (18), while in our study, multivariable logistic regression analysis showed that age groups were not related to IgM antibody results. However, recent travel history was associated with negative IgM. Cases with recent travel history were less likely to be IgM antibody negative [OR = 0.232, 95% CI: (0.128, 0.420), *P* < 0.001]. Asymptomatic cases had approximately twice the odds of being IgM antibody negative compared with symptomatic cases [OR = 2.583, 95% CI: (1.554, 4.293), *P* < 0.001].

**Table 3:** Multivariable logistic regression analysis for IgM and IgG.

	**IgM**				**IgG**			
	**OR**	**SE**	***P***	**95% CI**	**OR**	**SE**	***P***	**95% CI**
Gender	0.595	0.174	0.075	[0.336, 1.054]	1.345	0.371	0.282	[0.784, 2.310]
Recent travel history	0.232	0.070	<0.001	[0.128, 0.420]	0.986	0.273	0.959	[0.573, 1.696]
Infection type	2.583	0.669	<0.001	[1.554, 4.293]	0.511	0.145	0.018	[0.293, 0.891]
Age (years)
<20 (refer)
20–39	0.960	0.641	0.951	[0.259, 3.552]	0.389	0.189	0.052	[0.150, 1.009]
40–59	0.646	0.433	0.514	[0.173, 2.403]	0.364	0.180	0.041	[0.138, 0.959]
≥60	0.548	0.409	0.421	[0.127, 2.370]	0.417	0.236	0.122	[0.138, 1.264]

*IgG, immunoglobulin G; IgM, immunoglobulin M; SE, standard error; OR, odds ratio; CI, confidence interval*.

### Multivariable Logistic Regression Analysis for IgG

The antibody level of IgG antibody (positive or negative) was taken as the dependent variable, gender and infection type of infected person as the independent variables in Table 3. Female, cases without recent travel history and symptomatic cases were taken as the reference group. Different age groups were set as dummy variables, while the age group younger than 20 years was the control group. Multivariable logistic regression analysis showed that gender and recent travel history did not correlate to IgG positive results. Participants without symptoms were nearly less likely to be seronegative than those with symptoms [OR = 0.511, 95% CI: (0.293, 0.891), *P* = 0.018]. Convalescents aged 40–59 years were less likely to be IgG seronegative than those aged below 20 years [OR = 0.364, 95% CI: (0.138, 0.959), *P* = 0.041].

### The Duration of IgM and IgG

**Figure 1:**
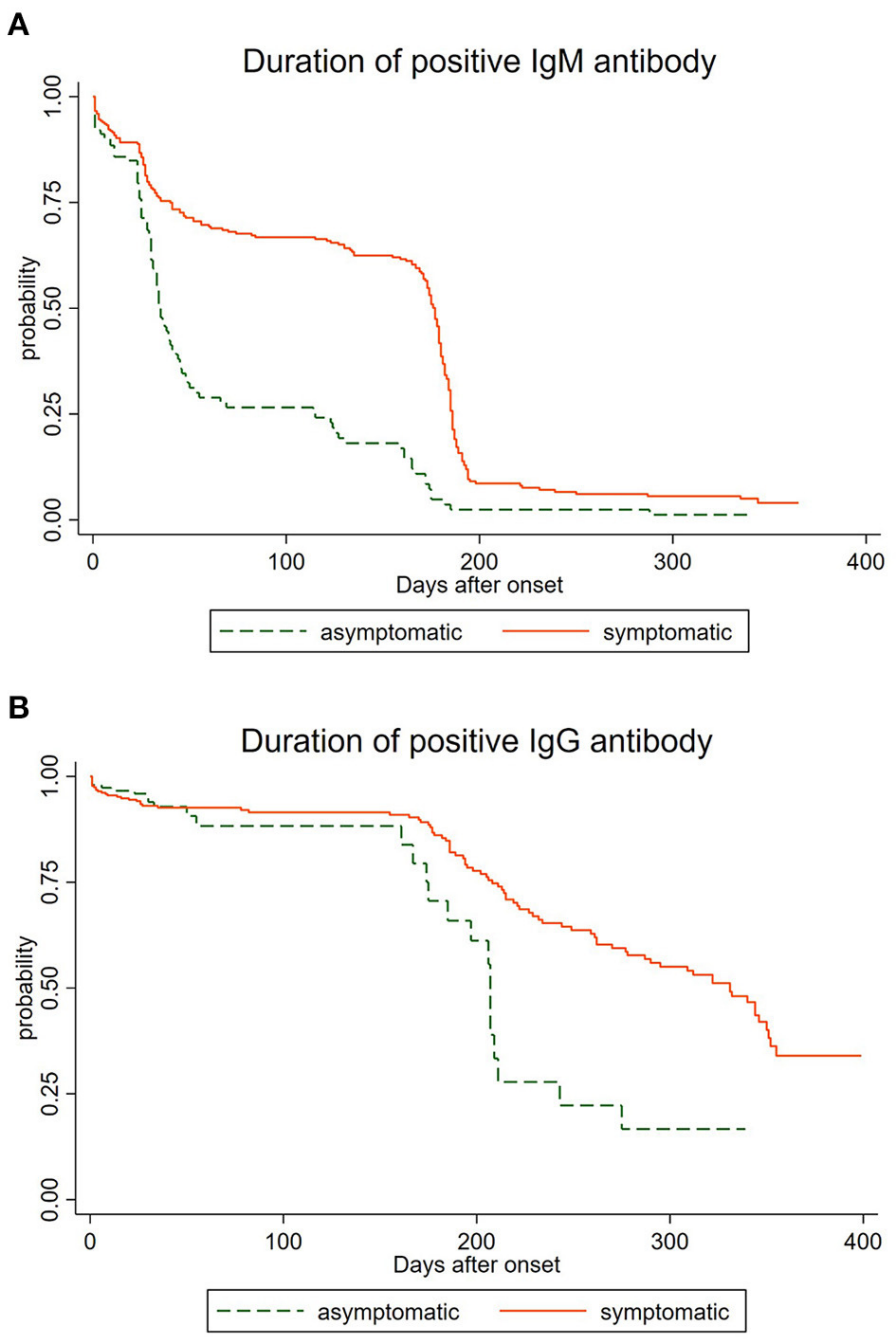
Duration of IgM and IgG antibodies among asymptomatic and symptomatic cases. **(A)** Duration of IgM antibodies among asymptomatic and symptomatic cases. **(B)** Duration of IgG antibodies among asymptomatic and symptomatic cases.

The analysis of the 1,707 serological samples showed that the total positive rate of IgG was higher than that of IgM during the whole study time, and the immune response persistence of IgG was longer than that of IgM antibody, which was consistent with the current research (17). We have observed that the longest duration of positive IgM antibodies was 365 days while the IgG persisted over 399 days, suggesting that there might be a long-term immune response after infection with SARS-CoV-2 (19). To comprehend the dynamics of antibody response, we regarded the occurrence of the negative antibody as a failure, and depicted the survival curve of differential antibodies in 517 convalescents. As shown in Figure 1A, positive rates of IgM antibody declined over time after natural infection with SARS-CoV-2, and IgG antibody prevalence decreased gradually after being infected (Figure 1B) for 150 days. We also observed a statistical difference between the asymptomatic group and the symptomatic group in the duration of positive IgM antibody (*P* < 0.05), as well as the duration of positive IgG antibody (*P* < 0.05). The total positive rates and the long-term duration of symptomatic cases were higher and longer than those of asymptomatic cases, as asymptomatic cases were more likely to be negative. However, the disappearance time of the two specific antibodies still needs further observation. We also found a statistical difference between the asymptomatic group and the symptomatic group (*P* < 0.05).

## Discussion

The human immune response is usually measured in the blood, and IgG and IgM antibodies are regarded as immune memory markers (20). Our study analyzed the serological outcomes from 517 convalescents of COVID-19 and associated factors of antibody response to SARS-CoV-2, which contributed practical information to the study of seroepidemiology of COVID-19. Our study suggested that the associated factors of being IgM antibody negative were recent travel history and infection type. In particular, recent travel history was associated with IgM. Cases with recent travel history were less likely to be IgM antibody negative [OR = 0.232, 95% CI: (0.128, 0.420)], probably because the virus has mutated to become more virulent and transmissible (21, 22).

Asymptomatic cases had approximately twice the odds of being IgM antibody negative compared with symptomatic cases [OR = 2.583, 95% CI: (1.554, 4.293)], which indicated that those who were asymptomatic required more attention, such as monitoring antibodies regularly and so on. On the contrary, we found that participants without symptoms were nearly less likely to be IgG seronegative than those with symptoms [OR = 0.511, 95% CI: (0.293, 0.891)], probably owing to the impact of mutations in SARS-CoV-2 on viral infectivity and antigenicity (22, 23).

As age was proved to be related to the results of the antibody (24), our study found that convalescents aged 40–59 years were less likely to be IgG seronegative than those aged below 20 years [OR = 0.364, 95% CI: (0.138, 0.959)]. We should pay attention to the cases of youngsters because these subpopulation lack sufficient protective antibodies to eradicate the virus. Meanwhile, we should monitor the convalescents without these protective antibodies considering they have a higher risk of getting re-infected (25).

We have observed that positive IgM antibodies persisted 365 days, while the IgG persisted for more than 399 days, which is of great significance for prevention and control. Over 90% of infected patients were tested to be seropositive and remained 120 days after diagnosis, suggesting their capacity to neutralize the virus (26). The duration of circulating IgG antibodies is still unclear and might depend on several factors, including the infection type and extent of immune response elicited upon the encounter with the virus (27).

As for differentiated characteristics, our findings did not cohere with previous researches. Though other studies indicated a sexual discrepancy in seroprevalence (28), we found no solid association between gender and SARS-CoV-2 immune response outcome, which may be explained by different innate immunity, steroid hormones, and factors related to sex chromosomes (29). Meanwhile, we observed that the duration of specific antibodies lasted more than 12 months, while the previous study reported 8 months (30), which suggested that long-term immunity existed in convalescents after natural infection. However, the duration still demands further surveillance.

We found that higher seroprevalence was present in patients aged between 20 and 60 years old, deviating from previous studies. For example, a previous meta-analysis revealed that a pooled SARS-CoV-2 seroprevalence in large population were 2.28% (1.01–3.56%), 3.22% (1.90–4.55%), 2.98% (1.59–4.36%), and 2.57% (1.39–3.76%) in people aged ≤19, 20–49, 50–64 and ≥65 years, respectively (10). Other studies showed that antibodies were often present in younger people (18–30 years old) (31), and that individuals younger than 50 years had a seroprevalence rate significantly higher than people older than 50 (32). There were several plausible explanations for such differences. First of all, different studies have different definitions of populations, some studies performed serological tests in the natural population (e.g., community) (33–35), but our study was conducted in recovered patients. Secondly, we have conducted multiple serum tests on the subjects, and a positive case was defined if any of the tests turned positive, while other large population studies conducted serum tests only once for screening (36–38), causing differences in age distribution. Thirdly, the diversity of the epidemic in different regions led to different infection conditions. Finally, due to public interventions in China, the elderly and children were mostly isolated at home, thus only a few cases in them. Furthermore, our study showed that the seroprevalence of convalescents aged 20–60 was higher, which was owing to their high mobility and proportion in patients.

As we define more clearly the natural immune response to SARS-CoV-2, its associated factors, and the duration of protective immunity, patient-centered practical guidelines will likely emerge. These tests may be useful for guarding public health, renewing risk management, and providing academic perspective, but additional data are required to fully drive this response (39). As the SARS-CoV-2 vaccine put a place in prevention, comparison of vaccine-induced immune responses to those stimulated by natural viral infection will help clarify immunological correlates of protection (16). These experiences from SARS-CoV are expected to provide implications for treatment, management, and surveillance of SARS-CoV-2 patients (40).

This research has several limitations. Initially, the research was expected to be based on continuous detection at different time points. While due to the lack of continuous observation on the data of patients, we have failed to report individual antibody responses continuously. Additionally, the individual's stabilization, after an initial drop in antibody levels and the inactivation time of specific antibodies generated by natural infection with SARS-CoV-2, still requires further tracking and testing. To this end, we expect this work will contribute to further long-term and continuous detection to investigate factors strongly related to serological levels and observe antibody dynamics over time, which may provide a deep insight into the immune response to SARS-CoV-2 convalescents and advance the development of vaccines and therapeutics.

Furthermore, we expect that serological study of SARS-Cov-2 convalescents during the recovery period would improve our understanding of the immunological response to SARS-CoV-2 infection, provide an auxiliary scientific basis for clinical development and evaluation of SARS-CoV-2 vaccine, and facilitate the continuous development of new vaccines and clinical therapeutics (41).

## Data Availability Statement

The datasets for this article are not publicly available because they will be used for further research. Requests to access the datasets should be directed to Xianping Wu, scjkwxp@163.com. The original contributions presented in the study are included in the article/supplementary material, further inquiries can be directed to the corresponding authors.

## Ethics Statement

This study was performed in compliance with all relevant ethical regulations and the protocol for human subject studies was approved by the Sichuan Center for Disease Control and Prevention (SCCDCIRB-2020-007). Written informed consent to participate in this study was provided by the participants' legal guardian/next of kin.

## Author Contributions

CL and LZ were major contributors to the formation of this manuscript, as they have consulted the literature, analyzed the data, and wrote the programs. HYu, XC, and CX also contributed to writing part of the manuscript. HYa, MP, and JX contributed to tests of serum samples and provided suggestions for this manuscript. XS, YZ, and JT input and cleaned up the data of specimen information. XD, HP, XC, TH, HL, DX, HW, WL, PZ, ZZ, and JL collected serums samples and delivered them to the laboratory. XW designed the serum antibody monitoring project and provided constructive suggestions. TZ contributed significantly to data analysis and manuscript preparation. All authors contributed to the article and approved the submitted version.

## Funding

This work was supported by the National Natural Science Foundation of China (Grant Numbers 82041033, 81602935), Sichuan Science and Technology Program (Grant Numbers 2020YFS0015, 2020YFS0091, and 2021YFS0001), Health Commission of Sichuan Province (Grant Numbers 20PJ092, 20ZDCX001), Chongqing Science and Technology Program (Grant Number cstc2020jscxcylhX0003), Central government funding items (Grant Number 2021zc02), LiangShan Yi autonomous prefecture (Grant Number H210322), and Humanities and Social Sciences Program of Sichuan University (Grant Number 2018hhf-26). The funding body did not participate in the design, collection, analysis, interpretation, and writing of this study.

## Conflict of Interest

The authors declare that the research was conducted in the absence of any commercial or financial relationships that could be construed as a potential conflict of interest.

## Publisher's Note

All claims expressed in this article are solely those of the authors and do not necessarily represent those of their affiliated organizations, or those of the publisher, the editors and the reviewers. Any product that may be evaluated in this article, or claim that may be made by its manufacturer, is not guaranteed or endorsed by the publisher.
